# An Uncommon Cause of Recurrent Dysphagia and Chest Pain in an Adolescent Boy

**DOI:** 10.7759/cureus.64648

**Published:** 2024-07-16

**Authors:** Ricardo Craveiro Costa, Joana Patena Forte, Marta Correia, Cristina Borges, Hugo Faria

**Affiliations:** 1 Pediatrics, Pediatric Hospital, Local Health Unit of Coimbra, Coimbra, PRT; 2 Pediatrics, Child and Adolescent Center, CUF Descobertas Hospital, Lisbon, PRT; 3 Pediatric Surgery, CUF Descobertas Hospital, Lisbon, PRT

**Keywords:** rare anatomical variants, gastrointestinal pathology, laparascopic surgery, rare cause of dysphagia, tubular duplication, gi development, esophageal duplication

## Abstract

An 11-year-old boy was brought to the emergency department with a week-long history of widespread pain in his upper abdomen that worsened with deep breathing and eating, sialorrhea, food impaction sensation, and a recent fever. Ten months prior, he had similar symptoms and was diagnosed with a pharyngeal phlegmon. He was treated with antibiotics and dexamethasone. In the current episode, he presented with mild elevation of inflammatory markers, a slight deviation of the trachea on chest X-ray, and a tubular esophageal duplication was identified on a thoracic CT, with its opening observed during the endoscopic study. The patient was admitted for further treatment with fluids, analgesia, and antibiotics, and showed improvement over the next seven days with no significant incidents. Esophageal duplications are a rare congenital anomaly and their exact cause is unknown. Typically found in the posterior mediastinum and lower esophagus, they can cause symptoms such as pain, dysphagia, regurgitation, and malnutrition. Surgical or endoscopic resection can be a treatment option for these malformations.

## Introduction

Esophageal duplications (EDs) represent a rare subset of congenital malformations characterized by their benign nature, a shared muscular wall with the esophagus, and an epithelial lining that corresponds to gastric mucosa in one-third to one-half of cases [1-4]. They are the second most common duplication of the alimentary tract after the ileum, comprising 10%-30% of cases [1,2,5-10]. Typically located in the posterior mediastinum, EDs can manifest as cystic or, less frequently, as tubular or diverticular forms, the latter being the rarest [1-3,5-7,10,11]. Tubular EDs are particularly rare, with an estimated incidence of one in 8,200 live births and a male predominance of 2:1 [4,5,7,12]. In contrast to the cystic forms, which correspond to the majority of cases (around 80%), tubular EDs often communicate with the normal esophagus [3,4,6,11].

The exact etiology of EDs remains elusive, but it is presumably attributed to the failure of vacuolization of the primitive septum between the fourth and eighth weeks of embryonic development. This consequently leads to the failure of the posterior primitive foregut to coalesce to form a single esophageal lumen [5,6,8,9,12-15]. Most EDs become symptomatic in early childhood and are therefore diagnosed before the age of two, or even in utero with the increased use of prenatal ultrasounds [1,3-9,12-14,16]. However, some EDs may not present symptoms until later in childhood or even adulthood [1,4-9,12,14]. When symptoms do occur, they can vary widely depending on the location and size of the duplication and may include dysphagia, chest pain, epigastric discomfort, regurgitation, vomiting, stridor, non-productive cough, and respiratory distress or recurrent pneumonia [3,5-10,12-17].

## Case presentation

An 11-year-old boy presented to our emergency room (ER) with one week of upper abdominal and lower thoracic pain exacerbated by deep breathing and eating. He reported sialorrhea and a long-standing sensation of "food impaction" during ingestion of both solids and liquids. Additionally, he had also developed a fever three days prior, which was well managed with ibuprofen every eight hours. He did not have any changes in his normal digestive pattern. He denied vomiting but mentioned frequent belching and occasional regurgitation. Physical examination was unremarkable except for persistent spitting of saliva, mild signs of dehydration, and a subjective impression of anxiety. Ten months earlier, he had experienced similar symptoms associated with severe odynophagia, dyspnea, and light dysphonia. Then, laboratory values showed a normal leukocyte count (14200/uL) with 75.3% relative neutrophilia and a C-reactive protein (CRP) of 5.88 mg/dL. Hemoglobin and platelet levels were normal, and a rapid strep test was negative. After evaluation by an ENT (ears, nose, and throat) specialist, a pharyngeal abscess was suspected, and the patient was admitted for treatment with intravenous dexamethasone and antibiotics (amoxicillin-clavulanic acid and clindamycin). A cervical CT revealed oropharyngeal stenosis, Waldeyer's ring hypertrophy, heterogeneous density in the parapharyngeal space, minor right-side tracheobronchial deviation, and parietal thickening of the hypopharynx and cervical esophagus. After clinical improvement, he was discharged four days later for outpatient therapy and a follow-up appointment with ENT.

**Figure 1:**
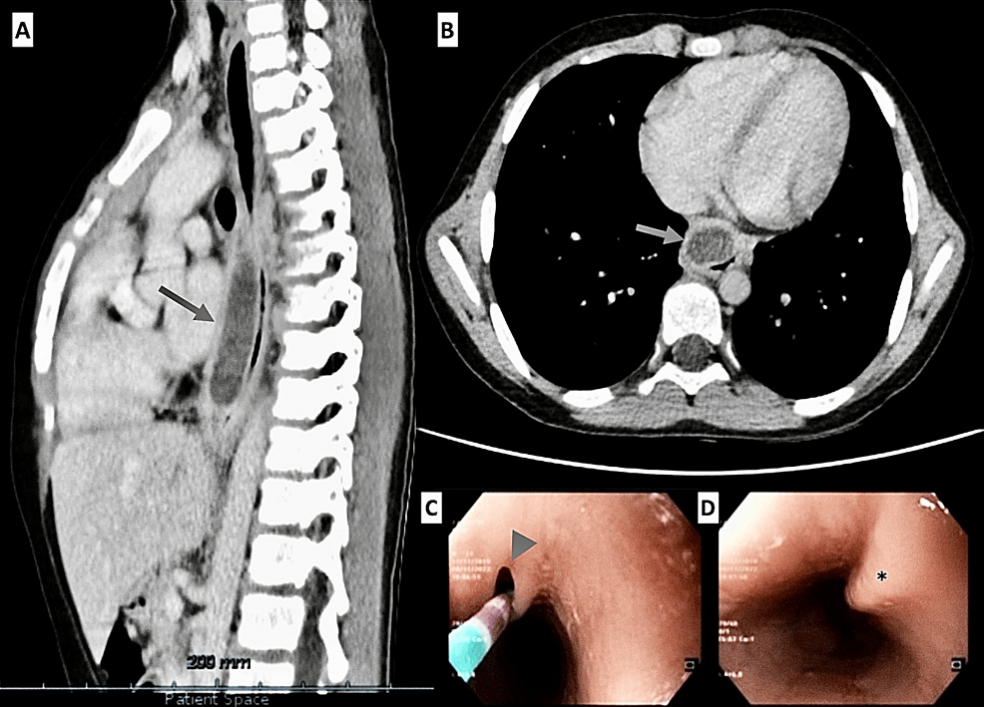
Imaging and endoscopic findings in tubular esophageal duplication Panels A and B: Thoracic CT showing a tubular formation with thick walls adjacent to the anterior thoracic esophagus, between the sixth and ninth thoracic vertebrae (arrow in panel A indicates the structure on a sagittal view, and arrow in panel B indicates the same formation on an axial view). The abnormality measures 6.5 cm longitudinally and 2.8 x 1.7 cm transversely, which was consistent with tubular esophageal duplication (TED) and mild upstream esophageal dilatation. Panels C and D: Upper digestive endoscopy revealing a well-preserved esophageal caliber with good distension. A 2 mm opening (arrowhead in panel C) at 21 cm from the dental arch spontaneously opened with insufflation. The remaining mucosa shows no lesions (asterisk in panel D).

On the most recent episode, a right-side tracheal deviation was again noted on the chest X-ray, prompting a thoracic CT to clarify a possible etiology. The CT revealed a tubular image with relatively thick walls next to the anterior flank of the thoracic esophagus, which was visible between the horizontal plane passing through the sixth and ninth thoracic vertebrae, corresponding to a maximum longitudinal diameter of 6.5 cm and about 2.8 x 1.7 cm in transverse and anteroposterior diameters, respectively. This finding was compatible with a tubular esophageal duplication (TED), and mild dilatation of the thoracic esophagus was recorded upstream (Figure 1, Panels A and B).

While in the ER, chest pain worsened, and an electrocardiogram was performed, showing a normal heart rhythm. Blood tests were ordered to check for potential bacterial infection, and the results revealed normal levels of hemoglobin and platelets, 12,600 white blood cells with 69% being neutrophils, a CRP of 5.87 mg/dL, urea of 45 mg/dL, and normal levels of creatinine and liver enzymes. The patient was then readmitted for further clarification of imaging results and received intravenous fluids, analgesia, and antibiotics (amoxicillin-clavulanic acid 150 mg/kg/day and clindamycin 40 mg/kg/day). The hospital stay was uneventful; the patient became apyretic, showing increased tolerance for food ingestion and improved dysphagia and sialorrhea.

An esophageal transit test (Video 1) revealed unobstructed contrast substance progression with a glove image in the distal half (approximately 5.7 x 1.5 cm). On the seventh day of admission, he underwent an upper digestive endoscopy, which described a well-preserved esophageal caliber, with good distension with insufflation, and a small opening of about 2 mm was identified at 21 cm from the dental arch, which opened spontaneously with insufflation. The remaining esophageal mucosa showed no apparent lesions (Figure 1, Panels C and D). After consulting with pediatric surgery, given the improvement in his clinical condition and the low risk of complications, as the paraesophageal structure had a good drainage orifice in the supine position, he could be discharged after completing seven days of intravenous antibiotic therapy, and an elective surgery via endoscopic approach would be later scheduled.

**Video 1:** Upper gastrointestinal (GI) series findings An esophageal transit test demonstrating unobstructed progression of contrast substance. A distinctive glove-shaped image, measuring approximately 5.7 x 1.5 cm, is visible in the distal half of the esophagus, indicating the presence of a structural anomaly.

An endoscopic approach was chosen as the best treatment option, involving the resection of the common wall through the opening in the upper segment of the tubular duplication. The patient successfully underwent the procedure without any complications and has remained completely asymptomatic following the surgery, with no additional symptoms or signs of recurrence. Regular follow-up appointments have confirmed the successful resolution of the condition.

## Discussion

EDs are rare congenital structures, with a prevalence of approximately one in 22,500 live births [17]. Some studies reported that they are seen in major pediatric referral centers at a rate of up to only three cases per year [7]. These malformations usually display a slight male predominance [4,5,7,12] and represent the second most common cause of posterior mediastinal masses in children, after neuronal tumors [17]. They are usually benign anomalies that arise from abnormal esophageal development due to a failure of vacuolization during the first weeks of embryonic development [5,6,8,9,12-15], resulting in a double layer of smooth muscle tissue enclosing a fluid-filled cavity that can be lined with varying types of gastrointestinal (GI) mucosa. Tubular EDs, which are even less frequent than cystic ones [1-3,5-7,10,11], can form along various parts of the esophagus, with a significant prevalence in the distal portion, accounting for two-thirds of cases [5,6,10,12-15]. In approximately 20% of these cases, the structure establishes a connection with the esophageal lumen, which can result in a broad spectrum of symptoms that impact both the GI and respiratory systems [3,4,6,11].

EDs are most commonly diagnosed in early childhood, but they can be found incidentally or remain asymptomatic until later in life, with fewer than 7% remaining asymptomatic until adolescence [1,3-7,9,11]. Symptoms can include dysphagia, chest pain, epigastric discomfort, vomiting, wheezing, stridor, and non-productive recurrent cough or recurrent respiratory infections, depending on their location [1,3-7,9,11]. Diagnosing EDs can be challenging and typically requires a combination of clinical, radiographic, and endoscopic findings [1,3,4,6-9,12,14]. Medical imaging tests such as X-ray, CT, or magnetic resonance imaging scans, along with barium swallowing tests and GI endoscopy, are employed to determine the size, location, and contents of the anomaly as well as to assess the patient's clinical status [1,3,4,7-9,11,12,14]. Once diagnosed, the next step is to determine the best course of treatment, which can range from conservative management with antibiotics to surgical intervention [1,3,4,7,12,15].

Treatment of EDs is controversial, especially in asymptomatic patients, with no clear established guidelines [3,6,14,17]. However, if symptoms appear at any time or depending on the specific characteristics of the patient, surgical intervention may be indicated [1,4,6-8,14]. Surgical resection is the standard management of EDs, typically involving the creation of a cleavage plane through the common muscular wall and repairing any defect if there is communication with the esophageal lumen [1,5,6,8-10,12-14]. The type of surgical procedure depends on the location and size of the ED as well as the patient's age and overall health status [1,5,8,12-14]. Minimally invasive techniques can fully remove some EDs, offering the advantages of faster recovery and less scarring [2,3]. In other cases, open surgical excision is necessary, particularly if the malformation is large or located in the lower part of the esophagus [1,5]. Recently, several reports have described successful treatments of tubular EDs through endoscopic lengthwise incision of the intraluminal bridge, resulting in short hospital stays and rapid return to normal oral intake [2,9,15,17].

Prompt and effective treatment of EDs is crucial to prevent complications such as infection, bleeding, mass effect, erosion, perforation, and the small but not negligible risk of malignant degeneration, thereby ensuring optimal outcomes [2-5,9,10,12-14,16,17].

## Conclusions

Although relatively uncommon, EDs are significant conditions to include in the differential diagnosis of chest pain and dysphagia in teenagers and young adults. Their clinical presentation and management can vary widely among patients. Effective management often requires a multidisciplinary approach involving accurate diagnosis, appropriate imaging, and, when necessary, prompt surgical intervention to avoid serious complications. Further research is needed to gain a better understanding of the prevalence and management of EDs in this demographic.

## References

[1] Soares R, Gasparaitis A, Waxman I, Chennat J, Patti M (2011). Esophageal duplication cyst. Dis Esophagus.

[2] Familiari P, Landi R, Mangiola F, Vita CV, Costamagna G (2020). Endoscopic management of a tubular esophageal duplication in a young adult. VideoGIE.

[3] Domajnko B, Salloum RM (2009). Duplication cyst of the sigmoid colon. Gastroenterol Res Pract.

[4] Oskayli MC, Ersoy F, Gulcin N, Pirim A, Ozel SK, Ozkanli S, Durakbasa CU (2022). Gastrointestinal tract duplications in children: a tertiary referral center experience. Medeni Med J.

[5] Saha AK, Kundu AK (2014). Tubular duplication of the oesophagus presenting with dysphagia. Singapore Med J.

[6] Sonthalia N, Jain SS, Surude RG, Mohite AR, Rathi PM (2016). Congenital esophageal duplication cyst: a rare cause of dysphagia in an adult. Gastroenterology Res.

[7] Khvorostov I, Gusev A, Alkhasov A, Yatsyk S, D'yakonova E (2022). Tubular duplication of the esophagus in a newborn, treated by thoracoscopy. European J Pediatr Surg Rep.

[8] Duan X, Cui Y, He Y, Xu S (2018). Acute attack of recurrent esophageal duplication cyst in an adult: case report and literature review. J Thorac Dis.

[9] Watanobe I, Ito Y, Akimoto E (2015). Laparoscopic resection of an intra-abdominal esophageal duplication cyst: a case report and literature review. Case Rep Surg.

[10] Tapete G, Ruggiero L, Gambaccini D, Marciano E (2023). A rare case of esophageal tubular duplication. J Gastrointestin Liver Dis.

[11] Spătaru RI, Lupuşoru MO, Şerban D, Ivanov M, Iozsa DA (2021). Alimentary tract duplications in children - a 15 years' experience. Rom J Morphol Embryol.

[12] Kolomainen D, Hurley PR, Ebbs SR (2017). Esophageal duplication cyst: case report and review of the literature. Dis Esophagus.

[13] Nayan S, Nguyen LH, Nguyen VH, Daniel SJ, Emil S (2010). Cervical esophageal duplication cyst: case report and review of the literature. J Pediatr Surg.

[14] Bagheri R, Asnaashari AM, Afghani R (2015). Esophageal duplication cyst. Asian Cardiovasc Thorac Ann.

[15] Okamoto T, Nakamura K, Ikeya T, Fukuda K (2021). Endoscopic fenestration with EUS guidance for esophageal duplication cyst. VideoGIE.

[16] Kapoor AK, Arora A, Haq S, Puri SK (2014). Esophageal duplication cyst. Indian J Gastroenterol.

[17] Garofalo S, Schleef J, Guanà R (2020). Esophageal duplication cyst in newborn. Pediatr Neonatol.

